# Cellular Senescence in Aging Lungs and Diseases

**DOI:** 10.3390/cells11111781

**Published:** 2022-05-29

**Authors:** Arbi Aghali, Maunick Lefin Koloko Ngassie, Christina M. Pabelick, Y. S. Prakash

**Affiliations:** 1 Department of Physiology and Biomedical Engineering, Mayo Clinic, Rochester, MN 55905, USA; aghali.arbi@mayo.edu (A.A.); pabelick.christina@mayo.edu (C.M.P.); 2 Department of Pathology and Medical Biology, University Medical Center Groningen, University of Groningen, 9713 GZ Groningen, The Netherlands; kolokongassie.maunicklefin@mayo.edu; 3 Groningen Research Institute for Asthma and COPD, University Medical Center Groningen, University of Groningen, 9700 RB Groningen, The Netherlands; 4 Department of Anesthesiology and Perioperative Medicine, Mayo Clinic, Rochester, MN 55905, USA

**Keywords:** aging, senescence, lung diseases, asthma, COPD, pulmonary fibrosis, remodeling, mitochondrial dysfunction

## Abstract

Cellular senescence represents a state of irreversible cell cycle arrest occurring naturally or in response to exogenous stressors. Following the initial arrest, progressive phenotypic changes define conditions of cellular senescence. Understanding molecular mechanisms that drive senescence can help to recognize the importance of such pathways in lung health and disease. There is increasing interest in the role of cellular senescence in conditions such as chronic obstructive pulmonary disease (COPD) and idiopathic pulmonary fibrosis (IPF) in the context of understanding pathophysiology and identification of novel therapies. Herein, we discuss the current knowledge of molecular mechanisms and mitochondrial dysfunction regulating different aspects of cellular senescence-related to chronic lung diseases to develop rational strategies for modulating the senescent cell phenotype in the lung for therapeutic benefit.

## 1. Introduction

**Figure 1:**
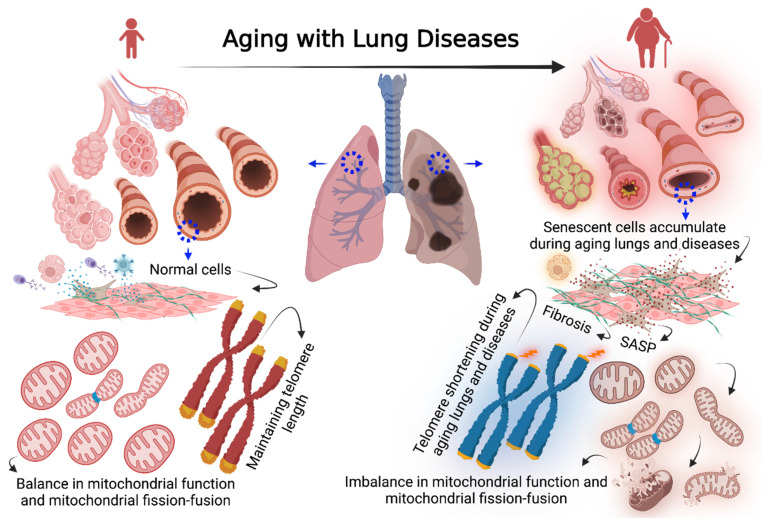
Left schematic figure shows a normal lung of young age with a low number of senescent cells rapidly cleared by immune cells, normal cells maintain a baseline of telomere length and mitochondrial homeostasis. Right schematic figure shows aged and diseased lung associated with increased fibrosis, higher numbers of senescent cells, and slow response of immune cells to clear senescent cells. Senescent cells are characterized by telomere shortening, secreting high rates of SASP, mitochondrial dysfunction, and an imbalance in mitochondrial fission and fusion. Figure 1 was created with BioRender.com accessed on 24 April 2022.

Cellular senescence is characterized by a permanent cell-cycle arrest triggered by various stimuli, including DNA damage to telomere shortening, genomic instability, epigenetic alterations, loss of proteostasis, and mitochondrial dysfunction (Figure 1) [1]. Despite being in cell-cycle arrest, senescent cells are resistant to apoptosis due to activation of anti-apoptotic signaling. Senescent cells remain metabolically active, secreting inflammatory cytokines, growth factors, chemokines (CXCs), and extracellular matrix (ECM) proteins, collectively known as senescence-associated secretory phenotype (SASP) [2,3].

Senescent cells are thought to have beneficial effects on repairing injured tissue and maintaining organismal integrity. The role of senescent cells in tumor suppression is also recognized. Under normal conditions, senescent cell burden is limited by removing excessive senescent cells via the immune system. However, with aging, impairment of the immune response results in accumulation of senescent cells that can exacerbate their effects leading to detrimental consequences, i.e., diseases of aging. Furthermore, there is now increasing evidence for different senescent cell phenotypes such that a shift towards detrimental, pro-inflammatory, pro-fibrotic senescent cells and SASP can occur with aging, contributing to disease.

Compared to other organ systems where senescence, SASP and contributions to aging and diseases of aging have been substantially explored, there is relatively less data on the aging lung, and senescent cells in aging-associated lung diseases such as COPD, pulmonary fibrosis, and even asthma. Senescent cells do accumulate in aging lungs and can exacerbate lung diseases [4,5,6] (Figure 1). However, the mechanisms by which senescent cells, via their SASP, can induce paracrine signaling to activate neighboring naïve cells to induce remodeling (altered ECM deposition and/or cell proliferation) or modulate cell–cell interactions to promote disease are still under investigation.

One factor relevant to cellular senescence and aging that may be of importance to the lung is cellular stress, which promotes mitochondrial dysfunction, including mitochondrial oxidative stress, mitochondrial DNA (mtDNA) mutation, imbalance in mitochondrial fission and fusion, and alterations in mitochondrial respiration [7]. Mitochondrial oxidative stress has been thought to be involved in accelerating aging effects. Separately, mitochondrial oxidative stress has been associated with lung diseases such as COPD and IPF and could thus play a role in stimulating as well as maintaining cellular senescence towards impaired lung function [8,9,10].

In this review, we discuss mechanisms of cellular senescence relevant to different aspects of the structure and function of aging lungs and to lung diseases, focusing on COPD and IPF. We review the influence of mitochondrial dysfunction in the context of cellular senescence and lung diseases. Finally, we summarize promising methods currently used to target senescent cells as a potential therapy to improve healthspan in the context of normal aging lung, and counteract lung diseases associated with aging. We appreciate that cellular senescence and SASP signaling is complex, and likely cell- and context-dependent. Accordingly, a review of these topics is necessarily brief and perhaps simple, but is relevant to the specific topic of aging lung and associated diseases. 

## 2. Overview of Cellular Senescence

Cellular senescence was originally described by Hayflick and Moorhead [11], where they demonstrated that human fetal fibroblasts lose their ability to divide after a certain number of subcultures (i.e., replicative senescence), associated with changes in cellular morphology such as flattening and increased cell size. Several studies have since described a similar phenomenon of replicative senescence in other cell types from different organs [12,13]. It is also now clear that other factors can accelerate cells towards losing their ability to proliferate in vitro, including the age and donor health condition [14], as well as environmental and genotoxic stresses. Examples include telomere shortening, inflammatory signaling, mitochondrial dysfunction and oxidative stress, oncogene-induced senescence (OIS), cell differentiation [15,16], chemotherapeutic drugs such as etoposide [17,18], exposure to UV radiation, and DNA damage stress.

Among cellular stressors, telomere shortening is now recognized as a hallmark of aging and senescence. Telomeres contain a repetitive nucleotide sequence of complementary double-strand DNA (5′-AGGGT-3′ and 3′-TCCCA-5) and wind up with a tail of a single-stranded DNA (5′-TTAGGG-3′) [19,20,21]. Telomeres protect chromosomal ends from recombination and fusion, and maintain DNA stability. Without telomeres, the DNA damage response is initiated [21]. In replicative senescence, the telomere shortens due to the inability of DNA polymerase to complete DNA replication. When a short length of telomeres is reached, a damage signal is initiated in the coiled DNA [22,23]. Therefore, telomere shortening has been used as a hallmark of aged and senescent cells [23,24,25].

Senescent cells are thought to support physiological functions during embryonic and postnatal development, tissue regeneration, and wound healing [26,27,28,29]. For instance, upon wound closure, activated myofibroblasts limit excessive fibrosis at the injury site [26,27,28,29]. The effects of senescent cells are kept in check by immune monitoring and clearance of senescent cells. Indeed, it is thought that during development, senescent cells avoid elimination from their microenvironment by altering their SASP components to avoid the immune system [26,27,28,29]. However, with aging, the efficiency of the immune system to clear senescent cells is impaired [30]. Consequently, senescent cells accumulate, secreting SASP factors that may become detrimental to naïve/neighboring cells by virtue of the quantity of such factors or an altered phenotype involved in more inflammatory and fibrotic elements [29,30,31,32,33].

## 3. Cellular Senescence Signaling Pathways

Cellular senescence is regulated by two signaling pathways that interact but are also independent: p53-p21^CIP1^ and p16^Ink4a^-Rb [18]. Permanent arrest of cell cycle occurs at the G_1_/S transitional phase distinguishing it from the quiescent phase, G_0_ [24,34,35,36]. The DNA damage response (DDR) regulates tumor suppressor of transcriptional factor p53 and downstream signaling p21^CIP1^, to result in permanent arrest in the cell cycle [18,24,34,35,36].

In the nuclei, DDR foci originate in response to DNA double-strand breaks (DSB). A subnuclear focus and accumulation of DDR proteins such as p53-binding protein 1 (53BP1), histone variant H2AX phosphorylated at serine-139 residue (γ-H2AX), and Ataxia Telangiectasia Mutated (ATM) in the vicinity of chromosomal DNA double-strand reflect early molecular events of cellular responses to DSB [34,37,38]. DDR then initiates a series of molecular events to repair DSB and to prevent potential DNA mutations. Phosphorylated at serine-139 in H2AX is mediated by ATM and Ataxia Telangiectasia and Rad3 related protein (ATR) kinases, which lead to visible DNA damage foci within the chromatin [18]. p53 binding protein is a key modulator rapidly localized to DNA damage foci after, for instance, ionizing radiation that causes DSB [37,38,39,40,41]. Although the key functions of p53 have not been fully understood, accumulated evidence suggests that the roles of p53 binding protein are engaging DSB proteins, such as interferon regulatory factor 4 (also known as MUM1) [35,38] and RAP1-interacting factor 1 (RIF1) [38,42], amplifying ATM activity, and promoting checkpoint signaling in response to low levels of DNA damage signals [38,39,40,43,44,45].

ATM and ATR stabilize p53 by activating cyclin-dependent kinase inhibitor p21^CIP1^, which in turn inhibits cyclin-dependent kinases-2 (CDK2) [33,39,40,43,46]. CDK2 triggers family members of tumor suppressors, retinoblastoma proteins (Rb), stopping the cell cycle in the S phase, and subsequently preventing DNA replication [29,31,43,47]. The signaling pathway of ATM-p53-p21^CIP1^/Rb results in a permanent arrest in the cell cycle [29,31,35,43].

Another tumor suppressor that influences key roles during cessation of cell division is the *INK4a-ARF-INK4b* locus [29,41,45,46,48,49]. The *INK4a* and *INK4b* locus encode for two cyclin-dependent kinase inhibitors, p16^ink4a^ and p15^ink4b^, while *ARF* is associated with p14^ARF^ in humans (p19^ARF^ in mice) [32,45,46,48,49]. *INK4/ARF* activates cyclin-dependent kinase inhibitor p16^ink4a^ that selectively inhibits cyclin-dependent kinases-4 (CDK4) and cyclin-dependent kinases-6 (CDK6) [29,31,32,43]. Upon activation, CDK4/6 phosphorylates retinoblastoma protein (Rb). As a result, transcriptional factor E2F3 is upregulated and leads to cell cycle arrest in the S phase [29,31,32,43,50,51]. Although upregulation of p16^ink4a^ is meditated by the downstream signaling of p53-p21^CIP1^ [29,31,32,43,52], it is believed that the transcriptional factor p21^CIP1^ upregulates earlier than p16 ^ink4a^ [47], giving a chance for cultured cells to go for another division cycle before making it to a complete cell cycle arrest [29,31,32,43,51,53]. Thus, the expressions of p53-p21 and p16 ^ink4a^ appear to demonstrate a non-linear functional relationship. 

## 4. Biomarkers of Cellular Senescence 

Accumulation of senescent cells can be recognized by utilizing various methods in vitro and in vivo. For instance, upregulation of the transcriptional factors p53, p21, and p16, and SASP elements such as IL-6 and IL-8, are well-validated markers [31,32,33,41]. Senescence-associated β-galactosidase (SA-βgal) is another technique that is widely used to identify senescent cells in vitro and in vivo [28,50], where due to increased levels of lysosomal enzyme, the enzymatic activity of SA-βgal results in blue color at a pH of 6.0 [50,52,54]. However, SA-βgal is not the most sensitive or specific marker of senescence. Fluorescence in situ hybridization (FISH) of telomerase combined with immunofluorescence staining of γ-H2AX results in localization of telomere-associated foci (TAF) and has more recently been used to identify senescent cells [22,24,28,55]. SASP and SASP regulators are also used to characterize senescent cells, including (1) proinflammatory factors such as IL-1α, and IL1ß, IL-6 and IL-8; (2) signaling pathway such as Akt and MAPK; (3) NF-kB [51]; (4) growth factors such as TGF-β1 and matrix-degrading enzymes, metalloproteinases; (5) extracellular matrix proteins such as fibronectin [47]. However, it should be noted that the SASP profile is highly cell and context dependent, and it is not unusual for the profile to change with time, making it difficult to identify a unique, stable, and broadly applicable set of senescence markers. In this regard, while RNA sequencing and whole-genome analysis have been widely utilized to identify senescence-associated genes [47,53,56,57,58], there is substantial interest in the use of fluorescence-activated cell sorting (FACS) and particularly cytometry by time of flight (CyTOF) using antibodies that recognize antigens selectively expressed in senescent cells and can distinguish between detrimental and beneficial phenotypes based on expression of p16 and p21 (generalized markers) and that of NF-kB (detrimental) [55,59]. 

## 5. Senescence Signaling in Lung Diseases 

**Figure 2:**
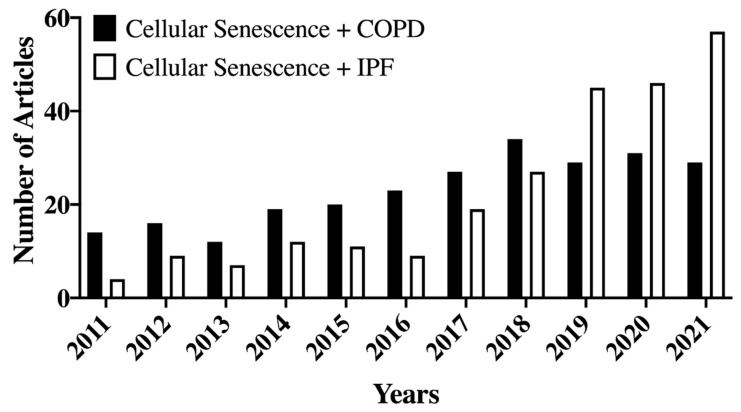
An increased number of articles related to Cellular Senescence and COPD or IPF have been published in PubMed-indexed journals during the past 10 years. Keywords used in PubMed search engines are “Cellular Senescence Chronic Obstructive Pulmonary Disease” or “Cellular Senescence Idiopathic Pulmonary Fibrosis”. Figure 2 was created from National Library of Medicine (https://pubmed.ncbi.nlm.nih.gov accessed on 24 April 2022).

Given the clinical impact of aging per se, and that of aging-associated lung diseases, it is important to identify biomarkers and signaling pathways in the context of senescence and its contributions to COPD [24] and IPF [60,61], and even asthma [18,62]. The importance of this area is reflected by the increasing number of research and review papers published on samples from human COPD and IPF patients and in animal models (Figure 2).

Lung tissues from patients with COPD and IPF show hallmarks of senescent cells [24,35,63,64]. Key biomarkers of senescence in aging adults are upregulation of p53, p21^CIP1^, p16^ink4a^, a robust release of SASP, positive staining for SA-βgal, TAF, and upregulation of anti-apoptotic signaling networks [24,25,35,63,65,66,67,68,69]. Increased expression of proinflammatory cytokines such as MCP-1, KC, MIP-1α, IL-12p40, and G-CSF have been observed in a mouse model of COPD [64,70]. In ROS-induced human senescent fibroblasts, IL-6 and IL-8 are increased following 14 and 25 days in culture [24,26].

## 6. Cellular Senescence in COPD

COPD is a major healthcare issue with a high morbidity and mortality rate [71]. COPD is characterized by obstruction in small airways (bronchiolitis), alveolar emphysema, and airway remodeling. Although tobacco smoke is the leading cause of COPD [63,72], air pollution, genetic disorders (alpha-1 deficiency), and respiratory infections are also risks for COPD. While there is no known cure, COPD is managed via lifestyle changes and medications, but these therapies have limitations, necessitating exploration of novel therapies.

Studies have shown that endothelial colony-forming cells (ECFC) derived from COPD patients have increased expression of SA-βgal, p16, p21, and γ-H2AX compared to ECFC isolated from control group patients [73]. In addition, lung fibroblasts derived from COPD patients show greater release of IL-6 and IL-8 and a higher percentage of SA-βGal staining [74]. Increased p21 and p16-positive epithelial cells have also been reported in COPD lung tissues compared to control groups [75]. More recently, COPD lung fibroblasts have been found to show senescence and 42 SASP secretome elements, which are implicated in chronic inflammation of COPD [76].

Tobacco smoke can trigger cellular senescence via oxidative stress-mediated DNA damage. Conversely, targeting p16-positive cells can inhibit tobacco smoke-induced emphysema in mouse models [63]. Furthermore, tobacco smoke accelerates telomere erosion and causes oxidative damage in cells [77]. Increased production of ROS associated with oxidative stress and changes in mitochondrial complex II, III, and V expression enhance cellular senescence [20]. Increased senescence in airway epithelial cells of severe COPD patients along with increased SASP has also been observed [3,78,79], and is relevant given the role of inflammation in COPD.

Multiple senescence signaling pathways may be involved in COPD, and activated in patients with a history of tobacco smoking and/or E-cigarette vaping [9,24,63,80,81]. For example, in older COPD patients, phosphoinositide-3-kinase (PI3K)-Akt and p38 MAPK cascades are activated [82,83]. Oxidative stress in COPD inhibits PTEN phosphatase activity, which in turn activates downstream signaling of PI3K-Akt and of mammalian target rapamycin complex 1 (mTORC1) protein kinase, which is a key player in cellular senescence. mTORC1 can also be activated by AMP-activated protein kinase (AMPK), an energy sensor that responds to an imbalance between AMP:ATP and ADP:ATP ratios [84,85,86,87,88]. Although AMPK is best known for its roles in cellular metabolism [78], its signaling is also important in the regulation of mitochondrial biogenesis and mitophagy [79,85,86,87,88]. Sirtuins, proteins involved in metabolic activity, have been implicated in aging and COPD lungs [80]. For instance, activation of mTOR upregulates microRNA-34a (miR-34a), and in return inhibits sirtuin-1 (SIRT1) and sirtuin-6 (SIRT6) activities [81,89,90]. Inhibition of SIRT1 dysregulates oxidative energy metabolism and influences NF-kB activity [91]. Activation of NF-kB stimulates SASP expressions found in many age-related diseases.

Another signaling pathway that plays a role in cellular senescence and COPD is p38 MAPK [92,93]. Increased p38 MAPK phosphorylation has been found in bronchial epithelial cells of COPD and asthmatic patients [92,93,94,95]. p38 MAPK signaling is known to enhance senescence burden in the lung [82,93]. SASP secretomes and oxidative stress stimuli such as tobacco smoke as well as respiratory pathogens can drive p38 MAPK phosphorylation in COPD lungs [90,93]. Increased p38 MAPK upregulates c-Jun proteins and activator protein-1 (AP-1), resulting in upregulation of microRNA-570 (miR-570), which inhibits SIRT1 and enhances NF-kB activity, leading to downstream activation of p53 and enhanced SASP expression [20,84].

Overall, these data provide evidence of senescence in COPD, and the potential involvement of multiple signaling pathways that could contribute to at least the inflammatory aspects of this disease. Of note, these signaling pathways are also well known to contribute to cell proliferation and remodeling and may thus be relevant to these aspects of COPD as well.

## 7. Cellular Senescence in IPF

IPF is a life-threatening chronic lung disease with poor prognosis and survival. IPF is characterized by scarred lungs associated with hyperproduction of ECM proteins [96,97]. In the past decade, there has been increasing interest in understanding the contributions of senescence to IPF (Figure 2). Several studies have shown that higher senescence markers are detected in IPF-derived cells and IPF tissues harvested from humans or in animal models. For example, upregulation of senescence-related pathways in alveolar type 2 (AT2) cells has been noted in a mouse model of IPF where AT2 Sin3a has been knocked out to induce senescence [61]. Conversely, targeting p53 signaling in alveoli reduces fibrosis [61]. p21 and p16-positive cells have also been shown to accumulate in IPF lung tissues [61,75]. Furthermore, SASP, such as matrix metalloproteinases MMP2 and MMP9 and collagen type I alpha 1 (COL1A1), show higher expression in IPF lungs [60]. Increased expression of p16 along with increased pro-fibrotic SASP has been reported in bleomycin-induced pulmonary fibrosis mouse models [60]. Sirtuins also play an important role during IPF as shown in fibroblast–myofibroblast differentiation (FMD), a process often triggered by TGF-β1. Reduced expression of SIRT-3 has been observed in IPF lung tissue, and inhibiting SIRT-3 has been associated with increased FMD in a murine IPF model after exposure to TGF-β1 [98]. Overexpression of SIRT-3 prevents TGF-β1-mediated FMD [98]. Thus, these limited data highlight the importance of senescence and associated signaling pathways in IPF.

## 8. Mitochondria in Senescence and Aging

Mitochondria are essential in eukaryotic cells for maintaining cellular homeostasis and function. Mitochondria regulate numerous cellular activities such as metabolism, replication, differentiation, senescence, and apoptosis [99]. Mitochondria produce energy for cells to perform essential functions by metabolized sources of macromolecules, such as glucose, amino acids, monosaccharides, and monoacylglycerols [99]. Several enzymes participate in the mitochondrial respiratory chain, a multistep process required to convert macronutrients into high-level energy. Mitochondrial respiration of glycolysis and the electron transport chain has been discussed in the literature extensively [100,101]. Herein, we will discuss mitochondrial roles in the context of senescence, aging lungs, and lung-related diseases.

Mitochondrial dysfunction can contribute to cellular senescence. For instance, gradual alterations of mitochondrial DNA (mtDNA mutations), variations in mitochondrial fission and fusion, elevated mitochondrial ROS production, and changes related to mitochondrial morphology (increased mitochondrial mass and elongation) can all play a role [35,102,103,104]. Senescent fibroblasts (replicative senescence) show dynamic changes in mitochondrial mass [26,28], while other studies show a dynamic feedback loop between damaged DNA and mitochondria [26,28,105,106].

Induction of senescence via disruption of mitochondrial function results in a distinctive SASP portfolio compared to senescence induced by genotoxic stress [107]. This mitochondrial dysfunction-associated senescence has been termed MiDAS and has been further shown to be a low NAD+/NADH ratio that drives it through AMPK-mediated p53 activation. Specifically, MiDAS secretomes are distinguished by higher levels of IL-10, CCL27, and TNF-α than core components of SASP such as IL-6 and IL-8 [107].

Overproduction of mitochondrial ROS is also an important player that causes DNA damage and results in DDR. The circle of ROS-DNA damage involves phosphorylation of DDR kinase ATM and Akt [108]. ATM activation initiates a series of phosphorylation events through ATM, NEMO, and IKK, ultimately activating nuclear transcription factor NF-kB, which enhances inflammation [108,109]. However, it is important to note that NF-kB activity is also affected by several factors, including metabolic activity and ROS production. For instance, reduced NAD+ and an alteration in AMP:ATP and ADP:ATP ratios affect SASP through NF-kB regulation [108]. Furthermore, activation of sirtuins such as SIRT1 and SIRT6 has been shown to inhibit NF-kB activity, affecting multiple SASP genes [100,110,111]. Activation of inhibitor sirtuins requires cofactor NAD+ [107,112]. Therefore, a reduction in the sirtuin cofactor NAD+ can increase NF-kB activity, and ultimately SASP responses.

Patients with COPD show changes in proteins that influence oxidative stress. PTEN-induced protein kinase-1 (PINK1), a mitochondrial stress protein marker that accumulates on the outer membrane of damaged mitochondria, is found to be elevated in COPD [72,113]. On the other hand, excessive production of mitochondrial catalase, an enzyme that protects cells from oxidative damage catalyzing hydrogen peroxide to oxygen and water, extends lifespan in the mouse [114]. Conversely, a reduction in prohibitin genes, such as PHB1 in the inner mitochondrial membrane that maintain mitochondrial function, has been observed in COPD and in smokers with no history of COPD [115,116]. Hydrogen peroxide can promote mitochondrial dysfunction in airway smooth muscle (ASM) cells [117,118,119,120]. ASM cells from patients with COPD show higher ROS associated with (1) increased IL-8 release, (2) decreased mitochondrial complex enzyme expression, and (3) reduced mitochondrial membrane potential [18,119].

## 9. Mitochondrial DNA Mutation in Aging Lungs and Diseases

Unlike the nuclear genome, the mitochondrial genome is a ~16.6 kb circular DNA molecule encoding subunits of polypeptides [99,105,106,121]. Diseases associated with mitochondria are driven by a variety of genetic mutations encoded by either the mitochondrial genome or nuclear genome [110,122,123]. Mammalian cells have multiple mitochondria, each having ~10 copies of DNA [106,121]. Mutation in mtDNA can be heteroplasmic or homoplasmic [106,121]. mtDNA is maternally inherited during embryonic development [111,124,125]. However, mtDNA mutations often occur during aging [82,126,127], where mutation rates are much higher in mtDNA than in nuclear DNA (nDNA) [111,128]. 

Emerging evidence illustrates that alterations of mtDNA are associated with electron transport efficiency. These changes are due to mutations in encoded subunits of polypeptides making up mitochondrial respiratory complexes that serve as primary sources of ROS. Depletion of mtDNA has been associated with premature aging and multiple chronic diseases [129]. Using a murine model, inducing mutation in mtDNA (by depletion) diminishes mitochondrial respiratory complexes I, III, and IV and ATP synthase [129]. These changes are associated with accelerated aging, skin hair loss, and increased inflammation [129]. Furthermore, introducing a deficiency in proofreading of mitochondrial DNA polymerase (POLG) (involved in mtDNA replication) results in premature aging in mice [71,130]. Studies show that introducing an error-prone version of mtDNA polymerase causes increased mtDNA mutation load [131] and a deficiency in mitochondrial respiratory complexes [131], and accelerates a premature aging phenotype in different mouse organs [71,131]. Other studies have reported that mutations in mtDNA are associated with aging and several chronic diseases [132,133,134,135]. For instance, much higher mtDNA mutation rates have been shown in Parkinson’s, Alzheimer’s, and cardiovascular diseases [128,136,137,138]. Furthermore, alteration in mtDNA reduces resistance to oxidative stress and increases risk of COPD, asthma, and other lung diseases [104,139,140]. More homoplasmic variants that lead to constant changes in electronic transport chain proteins have been observed in asthmatic patients [106]. Additionally, other studies have shown an imbalance between mtDNA and nDNA in asthma, COPD, and asthma–COPD overlap [141,142].

## 10. Cellular Senescence as a Therapeutic Target

Demonstrating that suppressing the accumulation of p16^Ink4a^ positive cells extends lifespan by decreasing growth hormone signals has helped to excite the field regarding the therapeutic potential of targeting senescent cells [126]. Subsequently, efforts to develop drugs that eliminate senescent cells (senolytics) without affecting normal cells have become a major focus in the field [68]. The idea is that senescent cells depend on their anti-apoptotic pathways to survive [68,132]. Senolytic cocktails of small molecules target the anti-apoptotic network [31,68,132]. Much work has focused on the use of dasatinib (D; a tyrosine kinase inhibitor) and quercetin (Q; a plant based flavonoid) [68,132]. D + Q were initially shown to effectively induce apoptosis in senescent cells of primary adipocyte progenitor cells and human umbilical vein endothelial cells but not in quiescent, proliferating, or differentiated cells [68,132]. In a mouse model, D + Q promotes physical function and reduces mortality in aged mice [143]. Human trials for D + Q in IPF suggest improvement in respiratory and physical function [62]. Recently, mice infected with SARS-CoV-2-related virus and treated with a senolytic showed reduced senescent cell burden and mortality while increasing antiviral antibodies [144].

Another approach to eliminating senescent cells is targeting the higher mitochondrial potential in senescent cells [145]. Mitochondria-targeted tamoxifen (MitoTam) is an anticancer agent that has been proven to inhibit oxidative phosphorylation and induce cell death in senescent cells [145]. MitoTam can selectively eliminate senescent cells in aging adults and premature or acute senescent cells at young ages [145].

**Figure 3:**
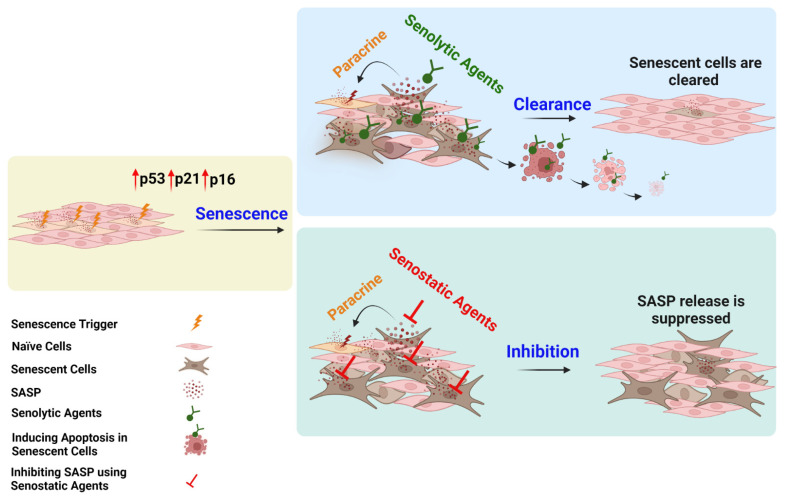
Shows different mechanisms between senolytic and senostatic agents targeting senescent cells. Left schematic figure shows normal cells exposed to DNA damaging agents resulting in upregulations of senescence signaling pathways. Top schematic figure shows senolytic agents selectively kill senescent cells in a living organism, inhibiting paracrine signaling with normal cell proliferation. Bottom schematic figure shows senostatic agents inhibit senescent cells releasing SASP, reducing paracrine signaling with normal cell proliferation. Figure 3 was created with BioRender.com accessed on 24 April 2022.

Besides senolytics, strategies to develop drugs that target signaling pathways critical to senescent cells have investigated using senostatic (senomorphic) drugs [69,127]. Unlike senolytics, senostatics (senomorphics) can block paracrine signaling that activates nearby naïve cells (Figure 3) [69,127]. While senolytics induce apoptosis and eliminate senescent cells, senostatics (senomorphics) are geared towards inhibiting SASP release and signaling and/or cell-specific SASP factor expression (Figure 3). Studies show polyphenols, flavonoids, and phytochemicals are effective senostatic drugs inhibiting signals and suppressing SASP factors [69,127]. Diminishing PI3K-Akt signaling via a prodrug pan-PI3K inhibitor CL27c in aging lungs decreases inflammation and improves life expectancy in murine animal models of acute or glucocorticoid-resistant neutrophilic asthma [133,146]. Additionally, activation of AMPK by reducing cellular metabolic activity and increasing ATP synthesis blocks mTOR activation. Consequently, the signaling cascade that enhances proinflammatory cytokine secretions and p53 activation is terminated. NF-kB antioxidants and inhibitors are effective senostatics, suppressing SASP factors [134,147]. Using rapamycin or torin to inhibit the mTORC1 signaling pathway has been shown to rescue mitochondrial dysfunction [135,148]. Similarly, the antioxidant drug MitoQ is effective at targeting TNF-α-induced CXCL8 [72,119].

Furthermore, the widely prescribed and FDA-approved anti-diabetic drug metformin shows promise in the context of senescence [149,150,151,152]. Metformin can activate AMPK by blocking complex I, which drives ATP synthesis in the mitochondrial respiratory chain, and thus improving the AMP: ATP balance [78]. These emerging data provide substantial promise to the idea of modulating senescent cell burden and/or SASP portfolios or downstream signaling towards alleviating lung diseases associated with aging.

## 11. Conclusions and Future Insights

Cellular senescence is a hallmark of aging lungs and aging-associated lung diseases. While senescent cells have beneficial roles, with aging, an enhanced senescent cell burden and a pro-inflammatory and pro-fibrotic SASP can contribute to pathophysiology of diseases such as COPD, IPF, and even asthma. Senescence can be activated by multiple upstream mechanisms, and conversely can involve multiple, interactive downstream pathways. SASP effects on naïve cells can be cell and context dependent with multiple effects on remodeling relevant to lung disease. Thus, there is substantial enthusiasm in exploring the use of senolytics and senostatics in eliminating senescent cells or modulating SASP effects towards therapy for lung diseases. Here, what remains to be understood is the differences in senescent cell and SASP phenotypes in different diseases, further complicated by likely cell-type differences in senescence in the lung. Appreciating that aging may differentially influence different cell types in the lung, the contribution of senescence remains to be understood in a cell-specific fashion. Thus, understanding the relative roles of resident cells such as bronchial and alveolar epithelium, smooth muscle and fibroblasts in senescence and its downstream impact is critical. In this regard, the relative roles of different senescence pathways may also show cell dependent variability that remains to be understood. Thus, modulation of senescence as therapy may be a reality for multiple aging-associated lung diseases; the potential for future research is high. 

## Data Availability

Links for data-generating Figure 2: Cellular Senescence Chronic Obstructive Pulmonary Disease https://pubmed.ncbi.nlm.nih.gov/?term=Cellular+Senescence+chronic+obstructive+pulmonary+disease&filter=years.2011-2021 (accessed on 22 March 2022). Cellular Senescence Idiopathic Pulmonary Fibrosis https://pubmed.ncbi.nlm.nih.gov/?term=Cellular+Senescence+Idiopathic+Pulmonary+Fibrosis&filter=years.2011-2021 (accessed on 22 March 2022).
